# Porcine parvovirus VP1/VP2 on a time series epitope mapping: exploring the effects of high hydrostatic pressure on the immune recognition of antigens

**DOI:** 10.1186/s12985-019-1165-1

**Published:** 2019-06-03

**Authors:** Ancelmo Rabelo de Souza, Marriam Yamin, Danielle Gava, Janice Reis Ciacci Zanella, Maria Sílvia Viccari Gatti, Carlos Francisco Sampaio Bonafe, Daniel Ferreira de Lima Neto

**Affiliations:** 1 Departamento de Bioquímica e Biologia Tecidual, Universidade Estadual de Campimas (UNICAMP), Rua Monteiro Lobato, 255, Cidade Universitária Zeferino Vaz, Campinas, SP 13083-862 Brazil; 2 Departamento de Genética, Evolução e Bioagentes, Instituto de Biologia, Universidade Estadual de Campinas (UNICAMP), Rua Monteiro Lobato, 255, Cidade Universitária Zeferino Vaz, Campinas, SP 13083-862 Brazil; 3 Embrapa Suínos e Aves, Laboratório de Virologia de Suínos, Concórdia, SC 89715-899 Brazil

**Keywords:** Epitope mapping, Epitope prediction, Porcine parvovirus, Spot synthesis

## Abstract

Porcine parvovirus (PPV) is a DNA virus that causes reproductive failure in gilts and sows, resulting in embryonic and fetal losses worldwide. Epitope mapping of PPV is important for developing new vaccines. In this study, we used spot synthesis analysis for epitope mapping of the capsid proteins of PPV (NADL-2 strain) and correlated the findings with predictive data from immunoinformatics. The virus was exposed to three conditions prior to inoculation in pigs: native (untreated), high hydrostatic pressure (350 MPa for 1 h) at room temperature and high hydrostatic pressure (350 MPa for 1 h) at − 18 °C, and was compared with a commercial vaccine produced using inactivated PPV. The screening of serum samples detected 44 positive spots corresponding to 20 antigenic sites. Each type of inoculated antigen elicited a distinct epitope set. In silico prediction located linear and discontinuous epitopes in B cells that coincided with several epitopes detected in spot synthesis of sera from pigs that received different preparations of inoculum. The conditions tested elicited antibodies against the VP1/VP2 antigen that differed in relation to the response time and the profile of structurally available regions that were recognized.

**Electronic supplementary material:**

The online version of this article (10.1186/s12985-019-1165-1) contains supplementary material, which is available to authorized users.

## Introduction

Porcine parvovirus (PPV), or Ungulate Protoparvovirus 1 as proposed by Cotmore et al. [1], is a 25-nm diameter, non-enveloped icosahedral virus that contains ~ 5 kb of negative sense, single-strand DNA (ssDNA) with two large open reading frames (ORFs) in its genome. ORF1 codes for the nonstructural proteins NS1, NS2 and NS3, and ORF2 codes for the structural proteins VP1, VP2 and VP3 [2]. VP1 and VP2 capsid proteins are the result of an alternative splicing of the same gene and VP3 is formed by proteolytic cleavage of VP2. These structural proteins are responsible for the immunogenic properties of PPV [3]. PPV infects pregnant gilts and sows, causing reproductive failure characterized by embryonic and fetal death, mummification and stillbirths, with delayed return to oestrus [4]. The resulting reduction in reproductive capacity can significantly decrease pork production [5].

PPV is prevalent in the pig population and highly stable in the environment, which make it difficult to establish and keep breeding populations free of the virus. For this reason, it is important to maintain herd immunity against PPV [4]. The most widely used approach for maintaining immunity is regular vaccination of breeding females. Two main strains of PPV, NADL-2 (non-pathogenic) and Kresse (pathogenic), have been identified based on pathogenicity, although sequence analysis of recent isolates suggests active evolution of PPV [6]. The vaccines commercially available since the 1980s are based on a chemically-inactivated NADL-2 strain.

The development of vaccines is important for controlling diseases such as reproductive failure caused by PPV. In this context, essential considerations should include viral diversity, protective immunity and population coverage as key factors, all of which pose special challenges to this ever-expanding field [7, 8]. One well-known approach in vaccine production is the use of inactivated viral preparations [9, 10]. In recent years, the use of high hydrostatic pressure (HHP) has become an increasingly popular non-thermal method of inactivation [11–13]. This approach has been successfully used to inactivate several viruses and represents a promising alternative to vaccine development [14]. A major advantage of viral-inactivation by HHP is that key immunological sites are retained intact, thereby enhancing the usefulness of the inactivated virus in vaccine development. HHP also induces a potent and directed immune response without introducing new chemicals to the process. In this study, we used a combination of in silico and in vivo approaches to examine the immune responses to different HHP-PPV formulations that were then compared with a commercial vaccine produced using chemically-inactivated virus.

## Material and methods

### Virus preparation

The NADL-2 strain of PPV was cultured in SK6 cells as previously described [15]. Supernatants of cell cultures were separated and purified by CaCl_2_ precipitation followed by gradient ultracentrifugation [16]. Fractions with hemagglutinating activity were dialyzed for 16 h at 4 °C against a Tris-EDTA buffer, pH 8.0 [17]. The virus was titrated in SK-6 cells by serial dilutions in 96 well-plates and the cells were then screened for a cytopathic effect at 72 h post-infection. The test was considered positive when cytopathic effects were observed in > 75% of the cells. Samples containing 1 × 10^4.5^ TCID_50_/mL were aliquoted and subjected to HHP. Vaccine aliquots and viral preparations were normalized to avoid an antigen-concentration effect [18].

Briefly, virus aliquots were subjected to two treatments: one at 300 MPa for 1 h at 25 °C and another at 300 MPa at − 18 °C, both immersed in 0.1 M Tris-HCl, pH 7.4. The equipment used to treat the samples consisted of a pressure generator device (model HP ISS) coupled to a pressure cell, as previously described [19]. The freezing point (i.e., − 19 °C) for these conditions was determined to be lower in previous studies by our group [20].

### Pig immunization and sample collection

This experimental study was done at the Embrapa (Brazilian Agricultural Research Corporation) Swine and Poultry Station, located in the southern Brazilian state of Santa Catarina. All the experimental protocols involving animals were approved by an institutional Animal Care and Use Committee (Embrapa, protocol no. 003/2009).

Ten 21-day-old specific-pathogen-free (SPF) male pigs from a terminal cross of Landrace and Large White lineages were divided into five groups of two pigs each: N (native PPV), P (PPV treated with 350 MPa at 25 °C), P-18 (PPV treated with 350 MPa at − 18 °C), V (commercial inactivated PPV vaccine, Farrow sure B, Pfizer) and NC (negative control). Pigs were vaccinated intramuscularly on day 0 (D0), D14, D28 and D38 according to each HHP protocol, as previously described [21]. Pigs in the NC group received only saline solution. On D51, all pigs were challenged intranasally with a reference strain of PPV NADL-2 [22]. Serum samples were collected prior to each vaccination or challenge (D0, D14, D28, D38 and D51), as well as on D58, D65 and D72.

### Spot synthesis

The peptides were synthesized based on the amino acid sequence of VP1 from PPV NADL-2 strain (GenBank, accession no. NC 001718) with 12 overlapping peptides and an offset of four peptides. The membrane used for spot synthesis was prepared with Fmoc (9-flurorenylmethoxycarbonyl) chemistry [23, 24] on a PEG-derived cellulose membrane with the addition of a C-terminal anchor of β-Ala residues and hexadecapeptides at the N-terminal. All serum samples from D0, D28, D58 and D72 (representing, respectively, the basal serum condition prior to any intervention, the antibody response against the vaccine, the challenge with the reference strain and 21 days after the challenge) were screened for antibodies. An anti-swine IgG-Fc secondary antibody conjugated with alkaline phosphatase (Jackson ImmunoResearch Europe Ltd.) was used to assess epitope recognition in the peptide arrays and downstream procedures were done as previously described [24]. The membrane was subsequently scanned at 1200 d.p.i. with a Print Scan Copier (HP Photosmart) and the software package “Totallab Quant-Array Analysis” was used to quantify the spots based on the intensity of the reaction relative to the background intensity. Signal below the arbitrary threshold of 0.2 were considered negative.

### Conformational epitope prediction

The complete primary sequence of the capsid protein of the reference strain was obtained from the UniProt Knowledge Base (UniProtKB) database (accession no. P18546) and used for downstream applications. The physicochemical properties of amino acids were considered and assessed using the Karplus and Schulz flexibility scale [25]. The antigenicity of the protein was inferred with the Kolaskar and Tongaonkar scale, a semi-empirical method to predict antigenicity from the physicochemical properties of amino acids in protein sequences [26, 27]. The hydrophobic properties of the sequence were predicted with the Parker hydrophilicity scale [28]. The full length VP1 protein has not yet been crystallized. Therefore, to obtain a better understanding of the epitope mapping results we produced models for the first 174 amino acids encompassing both the PLA_2_ domain and the nuclear localization signal, as well as the remaining residues, known to be buried in the PPV structure under physiological pH, but exposed in the early stages of productive infections. The phospholipase A_2_ (PLA_2_), nuclear localization signal and connecting regions of the VP1 protein were modeled using the software I-TASSER (v. 5.1) [29] and the output evaluated in the webserver MolProbity [30]. All models were energy minimized in GROMACS and protonation states were adjusted to pH 5.0 (to mimic those for endosome fusion states and PLA_2_ exposure). B cell epitope predictions were done using the crystal structure (PDB entry:1K3V) and the homology model generated for the VP1 complex with the softwares Bepro [31], Discotope2 [32] and Epitopia [33].

### Molecular dynamics

The crystallized structure of the VP2 protein (PDB code 1k3v) was downloaded from the Protein Data Bank and the coordinates were used to generate the VP1-PPLA model in the I-TASSER5.1 suite coupled with coordinates for the PPLA structure as the input for the homology model protocol (option 4). Molecular dynamics simulations were done with the GROMACS software (v. 2016.3) [34] to evaluate putative structural changes caused by HHP (350 MPa) at 27 °C and − 18 °C relative to the control conditions nder physiological conditions. Two systems were devised for the simulation run using a rombic dodecahedron as the simulation box limited at 10 A from the protein edges. The VP1-PPLA system was created with 138,811 atoms (11,221 atoms for the VP1-PPLA protein) and 75,857 atoms with 8447 atoms representing the VP2 solvated protein. The AMBER99SB force field was applied and the TIP3P explicit water model were selected for the nvt and npt equilibration protocols. The electrostatic interactions were calculated by using the particle mesh Ewald (PME) method for the van der Waals interactions and the electrostatic interactions in the real space of the PME method. Energy minimization steps were conducted in vacuum using the steepest descent integrator until convergence followed by solvation and ion equilibration of the systems to prepare the systems for the NVT and NPT equilibration steps. To simulate the experimental conditions the equilibration systems were normalized to 300 K at atmospheric pressure followed by modifications in the NVT and NPT mdp options to mimic the desired HHP conditions of 350 MPa at 27 °C and 350 MPa at − 18 °C. After the systems were equilibrated production runs for each condition were conducted for 20 ns and the outputs analyzed for structural parameters such as root mean square deviations (RMSD), root mean square fluctuations (RMSF), radius of gyration, secondary structure fluctuations and also for the complete system, such as pressure, temperature and energy variations. Plots were generated with the Grace software package (http://plasma-gate.weizmann.ac.il/Grace/) and trajectory analyses were done using the VMD package as well as the built-in analysis packages present in GROMACS [35].

## Results

### Epitope mapping

**Fig. 1:**
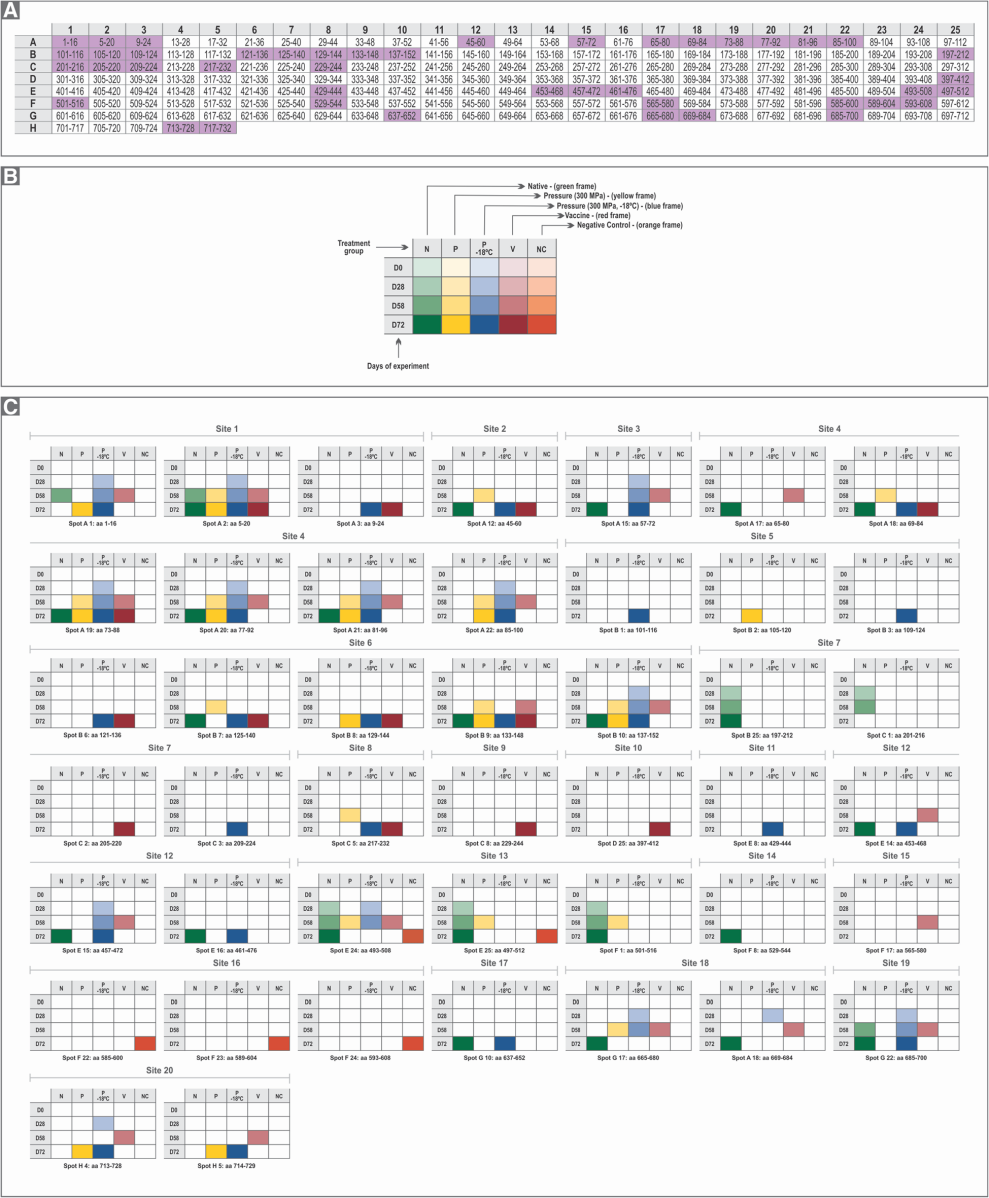
Epitope mapping results for the different conditions of pig inoculations. **a** Membrane spot array with each hexadecapeptide showing the VP1 amino acid position in each cell. The highlighted spots (purple, *n* = 44) correspond to positivity in at least one of the experimental conditions. **b** An explanatory framework for panel **c**. **c** Detailed data for the spots highlighted in **a**, showing the corresponding positivity for the experimental conditions and sample collection days (D0, D28, D58 and/or D72). Consecutive positive spots were denominated “sites” (*n* = 20)

**Fig. 2:**
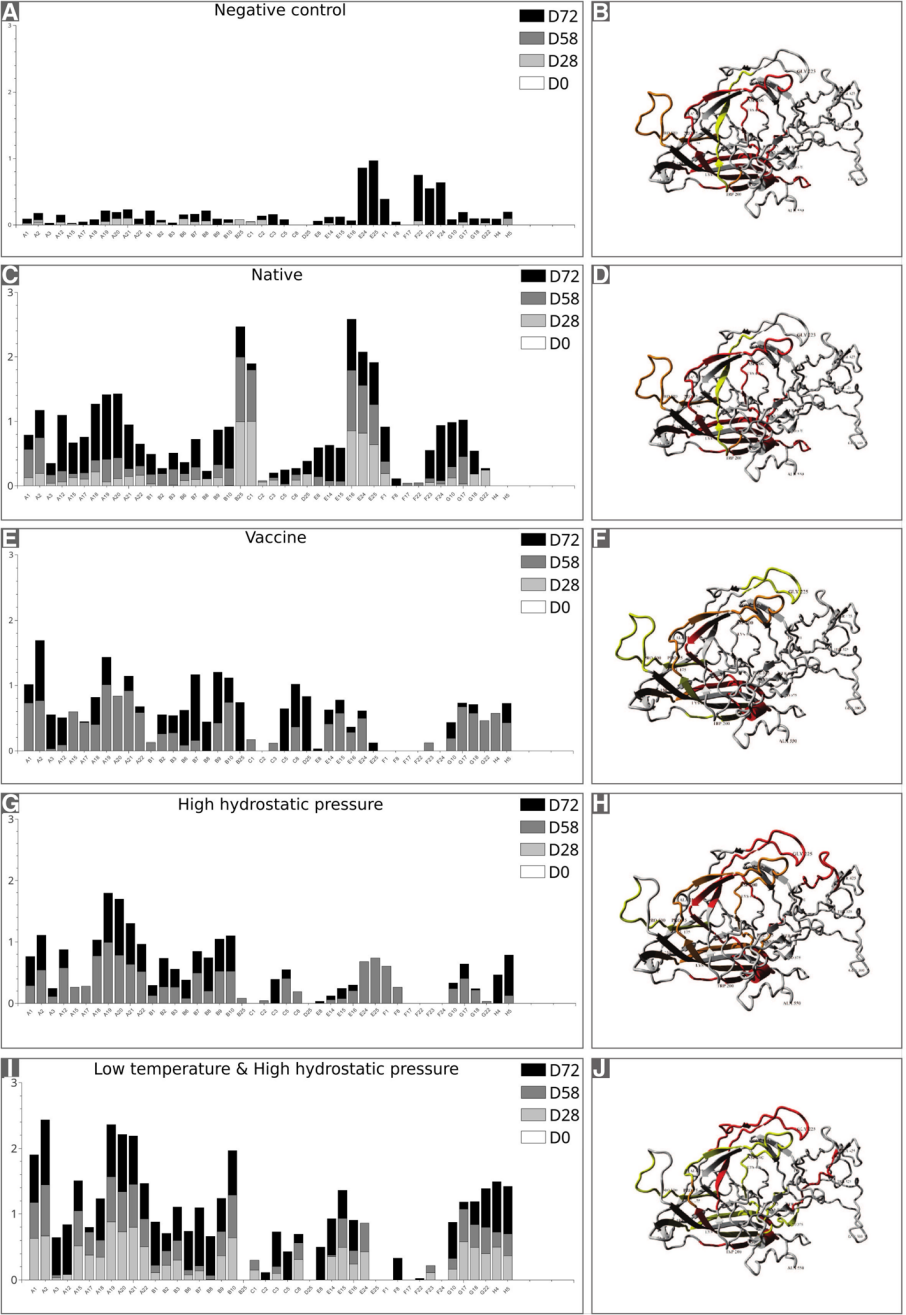
Intensity of the colorimetric output of the antibody-bound alkaline phosphatase reactions (panels **a** (NC), **c** (N), **e** (V), **g** (P) and **i** (P-18)) and a structural representation of each mapped region on the crystal structure of VP2 for each condition (panels **b** (NC), **d** (N), **f** (V), **h** (P) and **j** (P-18)). The signal intensity of the positive spots is shown in Fig. 1c, based on sample collection days D0, D28, D58 and D72, and was used to reconstruct epitope maps of PPV in graphs for the whole protein. The X-axis corresponds to the spot position (in agreement with Fig. 1) and four results are shown per spot. The Y-axis shows the relative intensity for each spot. The data was normalized for each experimental condition and a threshold was set at 40% of the maximum intensity. The models on the right show the crystallographic structure of VP2 protein (PDB entry 1k3v) with positivity indicated as yellow for D28 and/or D58, red for D72, and orange for positivity in both D28 and/or D58 and D72

A schematic representation of the membrane is depicted in Fig. 1a, with the positive spots highlighted in Fig. 1b and c, portraying the detailed conditions for each positive result (different HHP treatments and serum sampling intervals). None of the serum samples collected at D0 bound any polyclonal antibodies. Negative control pigs (group NC) showed positivity only after PPV inoculation (D58), which reflected sites usually activated when natural PPV infection occurs (sites 13 and 16 that correspond to amino acid residues 493–512 and 585–608, respectively) (Figs. 1c & 2a).

The data for each experimental condition and time point are shown as pairs of graphs in Fig. 2. The spot intensity is shown and was considered positive in Fig. 2a, c, e, f and i; the corresponding location was mapped onto the crystal structure of PPV VP2, with color coding according to the time point (Fig. 2b, d, f, h and j). There was a predominance of red and yellow regions in several conditions, indicating that the initial positivity in a given region frequently did not persist after challenge. In addition, spots that were positive after challenge were not preceded by positive epitope mapping in initial inoculations.

In comparison with the negative control the epitope mapping for the PLA_2_ domain in the VP1 sequence was pronounced in all treatments (Fig. 2a, c, e, g - Spots: A5 to A15 amino acids: 17 to 72). A similar pattern of immune response directed against these regions was observed in all treatments, differing mainly when the response was first detected. The PLA_2_ domain elicited a two-fold response when the P-18 treatment was used, suggesting that the treatment provided means for this region to be favored regarding antibody production at earlier stages if compared with the other treatments and positive control. The region formed by residues 61 to 124, associated with the connecting region between the PLA_2_ domain and the VP1 capsomer, produced higher and sustained antibody binding over the course of the experiments only for the P-18 treatment; the latter was detected in all data points after the first challenge, in contrast with the N, P, and V treatments for which the region began to be recognized only after D72 (Fig. 2a, c, e, g, Spots: A17 to B3, amino acids: 65 to 124). Overall, the low temperature and high pressure treatment induced prolonged antibody production with earlier availability for the first 174 residues spotted.

Pigs from group N showed no significant reaction on D28, except for sites 7 and 13 that correspond to amino acid residues 197–216 and 493–516, respectively. When samples from D58 (representing the immune response after PPV challenge) were analyzed, additional positivity occurred at sites 1 (aa 1–20) and 19 (aa 685–700). When antibodies obtained after the challenge with active virus were mapped against the membrane (D72), numerous additional epitopes were detected, namely, 2 (aa 45–60), 3 (aa 57–72), 4 (aa 65–96), 6 (aa 125–140; 133–152), 12 (aa 453–476), 14 (aa 529–544), 17 (aa 637–652) and 18 (665–684). The region initially detected at site 7 (aa 201–216) on D28 and D58 was not detected in the last sample (D72).

For group V, immunoreactivity was observed only on D58. In this case, PPV antibodies were detected at more sites than with the native form of the virus. Sites 3 (aa 57–72), 4 (aa 65–80; 73–100), 6 (aa 133–152), 12 (aa 453–472), 15 (aa 565–580), 18 (aa 665–684) and 20 (aa 713–729) had bound antibodies, in contrast to group N for which no signal was found in the same regions at D58. Epitope mapping after the viral challenge (D72) showed higher agreement with the map of group N, but distinct regions were also found for sites 1 (aa 9–24), 6 (aa 121–136; 129–144), 7 (aa 205–220), 8 (aa 217–232), 9 (aa 229–244) and 10 (aa 397–412).

In general, a higher number of sites was induced and recognized in group P than in group N, but this number was lower than in group V. Sites 1, 4, 6, 13 and 18 presented in group P were also found on D28 and D58 in group V, and sites 1 and 13 were also detected in group N. At the last sample collected (D72), the native form (N) showed more antigenic sites compared with groups P and V (Table 1S and Fig. 1c). Site 1 showed the highest frequency of positive spots on D28, D58 and D72 in groups N, P and V (Table 1S and Fig. 1c).

In group P-18, there was an increase in the number of positive spots (Fig. 1c). This situation was similar to group N, but the mapped response was higher when P-18 was compared to groups P, V and NC. Group P-18 pigs showed positive antibody responses earlier than the other treatments and included sites 1, 3, 4, 6, 12, 18, 19 and 20 (Fig. 1c). This group was the only in which sites 5 (aa 101–116; 109–124), 7 (aa 209–224) and 11 (aa 429–444) were activated on D72.

### Bioinformatic analysis

A combination of immunoinformatic tools was used to understand the molecular recognition of the VP1-VP2 capsid proteins based on the epitope mapping results described above. By using the reference sequence of NADL-2 strain, in silico predictions identified sites with a high probability of being antigens. Since this method does not evaluate tertiary structures and is based on a sliding window to produce propensity scores, we identified six regions out of the 24 predictions that overlapped with the results for group N.

Based on properties such as solvent-accessible surface area (SASA) and hydropathy predictions these regions were found to be exposed and hydrophilic, thus providing basic information for the experimental detection of such regions as epitopes in group N. In view of the conformational epitope predictions and conservative settings used, few regions returned positive conformational epitope predictions. However, by balancing for sensitivity and specificity the algorithms were able to detect putative epitopes on the 1k3v reference structure (public data), albeit in distinct regions when compared with our epitope mapping of the untreated virus.

Currently molecular dynamics simulations of viral proteins are common ground in viroinformatics, on the other hand using this tool to investigate HHP effects in combination with epitope mapping strategies is gaining momentum. Precious insights can be harnessed from molecular dynamics simulations and the correlation with our experimental desing and results added value to the in vitro results. The method is powerful enough to evaluate structural perturbations in a given system under pressure and it has been extensively used before to dissect the effects of HHP in proteins. As expected from previous reports our results show small deviations in RMSD fluctuations with decreased positional movements as a function of the conditions applied to the system in silico. Molecular dynamics analysis of the problem investigated the behavior of the VP1-PPLA (Additional file 1: Figure S1 & Additional file 3: Figure S3) and VP2 (Additional file 2: Figure S2 & Additional file 4: Figure S4) under normal conditions and under the conditions of groups P and P-18. A 20 ns molecular dynamics simulation allowed comparison of the radius of gyration (Rg), an indicator of protein compactness, with the group N simulation and revealed an overall decrease of 0.42% in Rg for group P vs. group N (2.85 nm vs. 2.838 nm) and a 0.87% decrease for the P-18 simulation compared to group N (2.85 nm vs. 2.826 nm) (Additional file 1: Figure S1 & Additional file 2: Figure S2), suggesting that the simulation produced a more constrained protein. The total energy of the HHP simulation increased 0.83% against group N (− 2.385 × 10^6^ cal versus − 2.405 × 10^6^ cal) whereas that for group P-18 increased 9.85% (− 2.385 × 10^6^ cal versus − 2.635 × 10^6^ cal) (Additional file 1: Figure S1). Conversely, a marked decrease in total volume was recorded during the group P and P-18 simulations compared with group N, which fluctuated around 1835 nm^3^ vs. 1680 nm^3^ (P) and 1655 nm^3^ (P-18), suggesting that the simulation was successful in creating the desired pressure and temperature for the experiment (Additional file 1: Figure S1 & Additional file 2: Figure S2).

The RMS fluctuation after the production run revealed the baseline values for the native protein atom fluctuations in accordance with the predicted flexible regions of the protein and provided a basis for comparison with the group P and P-18 simulations. As a result, the P simulation narrowed the amplitude variation per atom, thereby collapsing the flexible regions and limiting the overall variation to 0.01 nm. Furthermore, the P-18 simulation produced a more rigid configuration with no fluctuations > 0.05 nm on all atoms, in agreement with the other parameters (total volume, total energy and Rg). Interestingly, the area per residue over the trajectory increased substantially in the group P and P-18 simulations (Additional file 3: Figure S3 & Additional file 4: Figure S4). Moreover, the number of contacts maintained between residues decreased substantially when groups P and P-18 were compared with control group N (Additional file 3: Figure S3 & Additional file 4: Figure S4).

## Discussion

Epitope mapping strategies have been extensively applied to key pathogens and the results provided by this approach have important applications in public health, as well as in animal safety and welfare [36–40]. There are ongoing efforts to identify the key epitopes on the VP1-VP2 capsid proteins of PPV through distinct strategies for mapping. A study in which Pepscan was used to synthesize 24 different peptides directed towards antigenic sites detected nine such sites and posited that those found in the N-terminal region were neutralizing epitopes [41]. Xie et al. [3] confirmed these findings by using a monoclonal antibody (C4) against VP1 of PPV to screen a 12-mer phage peptide library. These authors identified a mimotope corresponding to the N-terminal immuno-dominant region that protected mice against the virus. In the present study, we mapped 20 “antigenic sites” corresponding to 44 positive spots based on a combination of experimental conditions that consisted to vaccination/challenge settings. Distinct patterns of epitope positivity were observed, depending on the experimental conditions to which the antigen was subjected and on whether the vaccination or challenge phase was being assessed (Fig. 2a-j).

To our knowledge, spot synthesis methodology has not previously been applied to PPV in this manner and the results obtained here provide a comprehensive landscape of the immunological profile of the VP1-VP2 complex by slicing the protein into overlapping oligopeptides bound to a nitrocellulose membrane. The spot results are quantifiable and give satisfactory parallel comparisons with ELISA findings, such as for determining protein concentration [42]; the methodology is reproducible and has been used for quality control [43].

The PLA_2_ domain is responsible for crucial steps in the PPV infection cycle by facilitating the release of the virus near the nucleus after successful endosome formation and prior to the activation of nuclear localization signaling. Numerous studies since the 1980s have investigated the latter event following the demonstration that microinjections of antibodies can interfere with intracellular antigens [44]. The inhibition of antigen function by the binding of antibodies to their antigen within endosomes is dependent upon factors such as exposure, identity and, primarily, pH. Important advances have been made using genetic engineered antibodies to sustain binding at lower pH [45]. Identification of the IgGs that successfully bind to key viral proteins or functional domains (as in the present case) is of utmost importance in tackling infections early on. The results described here indicate that the native conformation of the virus elicited an immune response against the PLA_2_ target, but only in later stages of antibody production, whereas pressure and the combination of low temperature and pressure evoked an exuberant response that was significant at all sampling intervals in the P-18 experiment.

By combining molecular dynamics with epitope mapping, it was possible to investigate the putative alterations that HHP may have induced in protein conformation and in the solvent-accessible surface area. The native form of the protein, used here as a positive control, provided the epitope landscape to which pigs responded at three sampling times. Initially, group N showed a strong antibody response for spots B25 (aa 197–212) and C1 (aa 201–216) at sampling times D28 and D58. These spots correspond to the N-terminal region of VP2, information that is also present in the crystal structure of VP2. Epitopes located in the VP2 capsid protein of PPV are important for generating neutralizing antibodies against this virus, with a high potential to activate B cells. These studies indicate that the N-terminal of VP2 is a strong candidate for B cell epitopes and should be included in vaccines [46, 47]. Antibodies that bound to a region composed of amino acids 497–516 were seen soon after the second inoculation and the titers (inferred from the intensity of the phosphatase reactions) were maintained throughout the experiment. The immunodominant regions in VP2 belong to the loops and other regions in the N- and C-terminals [41, 46, 47]. As shown in Fig. 2b, d, f, h and j, the loop regions marked by Pro^500^ and Val^175^ occurred in epitopes present in the five groups, with an alternation of these regions in three intervals of serum collection.

Although the capsid proteins were deemed to be under near-neutral selection, a few key amino acids were found to be variable (aa 215, 228, 383, 414, 419 and 436), with most of them located in surface loops [6, 48]. Zeeuw et al. [49] have previously shown that the strain switch from avirulent (143a) to virulent (27a) might depend directly on mutations of VP2 residues 378 and 383. As expected, surface amino acids in the VP2 configuration are hydrophilic and contain most of the mutations identified by Ren et al. [50], who also found this protein to be under negative selection. Antibody responses against overlapping peptides of VP1 were not detected until late time points, precisely after the viral challenge, when amino acids 5 through 96 produced significant binding, thereby reinforcing previous findings regarding the N-terminal immunogenicity of PPV VP1 [3].

In contrast to these results, vaccine epitope mapping elicited marked responses against the N-terminal region of VP1 after D38, with moderate binding to the previously mentioned VP2 regions. HHP elicited responses like those of the vaccine in the VP1 N-terminal region, as well as for the VP2 variable regions mentioned before on the fourth inoculation, prior to the challenge with PPV. In our study, amino acid residues (45, 217–219 and 556) corresponding to the mapped epitopes included some of the sites reported by Streck et al [51] The substitution of amino acid residues at sites located mainly on the surface of PPV is important not only for understanding the evolutionary mechanism of adaptive response to the host, but also for identifying possible strategies to produce more effective vaccines against PPV.

HHP treatment of PPV at 25 °C and − 18 °C did not suppress antigenicity but elicited the appearance of several other epitopes through the exposure of parts of the capsid. Although HHP did not directly affect the tertiary structure of the proteins, the exposure of different parts of the proteins, including hydrophobic regions, in response to these conditions could explain the appearance of new epitopes [52]. A direct comparison of the secondary structures of the VP2 crystal structure obtained after a 20 ns molecular dynamics simulation of the experimental conditions also showed small alterations to the original configuration (SF 2ary), mostly from switches between flexible regions (the most frequent interconversions found were from coil to bend, turn to3-helix and alpha-helix to turn to 3-helix). Conversely, HPP and low temperature HPP produced more stable secondary structures compared to the simulation with no pressure or temperature constraints. HHP and low temperature HHP simulations displayed small fluctuations during the production run and only the N-terminal region of the VP2 1K3V structure showed changes in configuration (amino acids 40–90). Compared with the molecular dynamics of native VP2, the HHP simulation tended to produce fixed turns where interconversion between bends and turns were recorded (amino acids 45–55) whereas low temperature HHP fixed this region in a 3-helix configuration. The region from amino acids 75–85 fluctuated among alpha-helix/turn/3-helix configurations in the native simulation but the region was then fixed as an alpha-helical structure in the HHP run and varied between turns and bends in the low temperature HHP run. The interactions mounted by the protein-solvent system at HHP are more favorable and due to small deformations in the internal bonds the protein-protein interaction energies are also increased (total energy graphs). The H-bond network is also favored by HHP, as previously reported for SOD, lysozyme and BPTI, the results for the capsid proteins H-bond network were increased during the simulation and were significant when compared against the N state. More studies are necessary to evaluate if possible correlations can be produced from MD simulations and putative immunological responses. An important limitation of our study is the simulation of single proteins in water, it is of particular interest full-atom simulations of complete viral capsids in water subjected to HHP and cold denaturation processes to expand in this field as computational power becomes available.

Previous reports have identified important structural changes in globular proteins after exposure to HHP, with increased disordered structures and turns, as well as decreased β-sheets and α-helices [13]. These findings suggest that the effect of pressure on kinetics arises from a larger positive activation volume for folding than for unfolding; this in turn leads to a significant slowing down of the folding rate with increasing pressure. For viral capsids under HHP, D’Andrea et al [53] noted that the treatment enhanced the viricidal effect on hepatitis A virus, a non-enveloped virus, indicating that the efficacy of treatment depends on capsid conformation. These authors suggested a correlation between mature and immature capsids, with the accessibility of the immunodominant site near the five-fold axis possibly explaining the susceptibility of the virus to inactivation by HPP [53].

Current methods of vaccine production make use of hazardous chemicals that may pose a risk to vaccine recipients. The efficacy of HHP for inactivating a variety of foodborne pathogenic microorganisms is well-established, and some of these microorganisms have been demonstrated to retain immunogenic properties, a finding which suggests that HHP may have an application in vaccine development. Indeed, pressure can result in virus inactivation while preserving immunogenic properties [11, 12, 14, 21]. The forces governing viral assembly and disassembly rely on both the microenvironment and the protein-protein interaction network produced by their genetic codes [54]. The contributions from studies on the dynamics of capsid autoassembly in vitro and in silico have provided interesting insights into the behavior of such ensembles. As illustrated by Silva et al. [14] and tested in silico in a number of conditions [55–57], the use of HHP methods alters the distribution of forces in the viral capsid to promote an energy compliant structure; this treatment results in a tangible output as epitopes revealed by this method are of great interest for both diagnostics and basic research.

More studies are necessary to understand the influence of the selective pressures on structural conformations and the correlation of the HHP method in revealing different epitopes. Recent work by Letko et al. [58] addressed fundamental questions on the subject by conducting serial passages of the virus while selecting for a new receptor adaptation expressed on the target cell; this approach allowed the identification of key structural aspects in the viral code that permitted receptor adaptation to a new receptor. In silico simulation methods provide good models for processes on this scale and allow important comparisons with experimental approaches, with all-atom simulations of full virus capsids becoming computationally feasible for most laboratories [59]. The latter authors also suggested that a dynamic h-bond network could potentially have a role in ion selectivity within the capsid. The effect of HHP on protein structure has been increasingly studied in silico as computational power and resources become increasingly available; such analyses have substantially advanced our understanding of the influence of each experimental condition on its target. Protein compressibility has been tested in silico in several configurations. Voichita [60] reported the effect of pressure on globular proteins by measuring the variations in protein total volume and the volume variation per residue after picosecond exposures. The findings suggested that protein volume was significantly compressed during HHP experiments in silico and that this effect was mostly due to the difference between SASA and the VdW radii. Secondary structures also contributed to the results obtained, with the compressibility of α-helices being inferior to that of β-sheets. In contrast, Vahidi et al. [61] found that in silico HHP failed to modify secondary structures such as β-barrels, thus agreeing with previous findings for loops and helical structures.

Inactivated or attenuated strains of PPV for vaccine production may be useful for controlling this virus in pigs. In this context, epitope mapping of immunogenic proteins of PPV is crucial for assessing the immune recognition of such viral preparations with a potential use in vaccine preparation, as shown here for the immunization of pigs. In the present case, the combination of HHP and epitope mapping provided a broader, more refined perspective of the epitope landscape stimulated by each preparation. This information could potentially be used to track HHP-vaccinated pig herds as a first scenario, or, conversely, be used as a combined alternative to cover the immune response against the virus. Taken together, our findings provide detailed insight into the immune system of pigs when the animals are stimulated with the same molecular configuration of an antigen, the structure of which can be altered by pressure and temperature. These data also improve our understanding of site-directed antibody production over time and of the putative immunological dynamics of antibody selection before and after a challenge with antigen.

## Additional files

Additional file 1: **Figure S1**. Results for molecular dynamics production runs: First line: results for 20 ns of a negative control production run for VP1-PPLA model. A – Volume, B – Temperature, C – Pressure. Second line: results for 20 ns of a production run for VP1-PPLA model simulated under pressure and low temperature. D – Volume, E – Temperature, F – Pressure. Third line: results for 20 ns of a production run for VP1-PPLA model simulated under pressure: G – Volume, H – Temperature, I – Pressure. (TIFF 4251 kb)

Additional file 2: **Figure S2.** Results for molecular dynamics production runs: First line: results for 20 ns of a negative control production run for 1k3v (VP2 crystal). A – Volume, B – Temperature, C – Pressure. Second line: results for 20 ns of a production run for 1k3v simulated under pressure and low temperature. D – Volume, E – Temperature, F – Pressure. Third line: results for 20 ns of a production run for 1k3v simulated under pressure: G – Volume, H – Temperature, I – Pressure. (TIFF 4032 kb)

Additional file 3: **Figure S3.** Results for molecular dynamics production runs: First line: results for 20 ns of a negative control production run for VP1-PPLA model (A,B – N; C,D – P; E,F – P-18). A – RMSF per residue, B – Solvent Accessible Surface Area per residue, Second line: results for 20 ns of a production run for VP1-PPLA model simulated under pressure and low temperature. C – RMSF per residue; D – Solvent Accessible Surface Area per residue. Third line: results for 20 ns of a production run for VP1-PPLA model simulated under pressure: E – RMSF per residue, F – Solvent Accessible Surface Area per residue. (TIFF 1123 kb)

Additional file 4: **Figure S4.** Results for molecular dynamics production runs: First line: results for 20 ns of a negative control production run for 1k3v (VP2 crystal) (A,B – N; C,D – P; E,F – P-18). A – RMSF per residue, B – Solvent Accessible Surface Area per residue, Second line: results for 20 ns of a production run for 1k3v (VP2 crystal) simulated under pressure and low temperature. C – RMSF per residue; D – Solvent Accessible Surface Area per residue. Third line: results for 20 ns of a production run for 1k3v (VP2 crystal) simulated under pressure: E – RMSF per residue, F – Solvent Accessible Surface Area per residue. (TIFF 1121 kb)

## Abbreviations

3D: Three dimensional
HHP: High hydrostatic pressure
HI: Hemagglutination inhibition
ID: Identification
IEDB: Immune Epitope Database
ORFs: Open reading frames
p.i.: Post-infection
PPV: Porcine parvovirus
R(g): Radius of gyration
RMSD: Root-mean-square deviation of atomic positions
RMSF: Root-mean-square fluctuation
SK6: Swine kidney cell
SPF: Specific pathogen free
TCID_50_/mL: Median Tissue Culture Infectious Dose
